# Supplementary material for: Proteomics of Duchenne Muscular Dystrophy Patient iPSC-Derived Skeletal Muscle Cells Reveal Differential Expression of Cytoskeletal and Extracellular Matrix Proteins
Source: Cells. 2025 Oct 28;14(21):1688. doi: 10.3390/cells14211688 (PMC12611083; doi:10.3390/cells14211688)
Supplement: Supplementary file 1 [file cells-14-01688-s001.zip › cells-3890693-supplementary.pdf]

cells

Supplementary Materials: The following supporting information can be downloaded at: https://www.mdpi.com/article/10.3390/cells14211688/s1,
Figure S1: Validation of myogenic identity of skeletal muscle differentiation in the 2D cell cultures and 3D organoids.
Figure S2: Volcano Plots_2D : WT vs. DMD1, WT vs. DMD2, WT vs. DMD3;
Figure S3: Volcano Plots_3D: WT vs. DMD1, WT vs. DMD2, WT vs. DMD3;
Table S1: Core Overlap;
Table S2: 2D quantifiable proteins;
Table S3: 3D quantifiable proteins;
Table S4: 3D significantly differentially expressed proteins;
Table S5: 2D significantly differentially expressed proteins;
Table S6: 2D GoTerm Analysis;
Table S7: 3D GoTerm Analysis

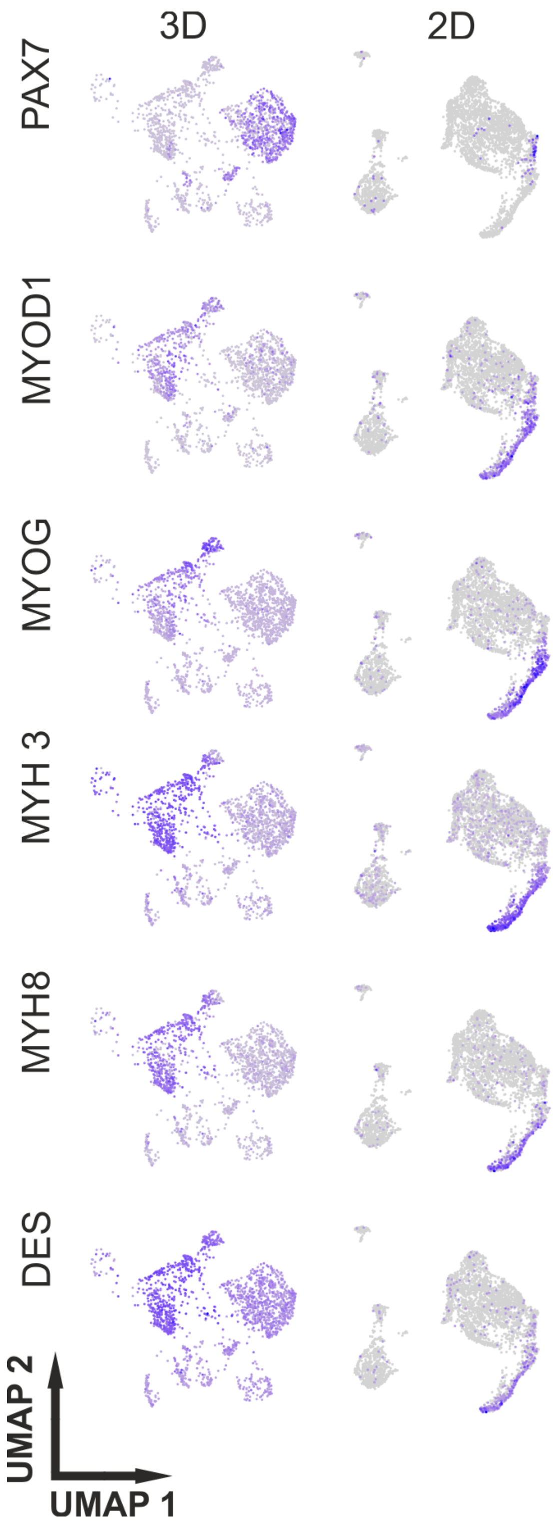

Figure S1 Validation of myogenic identity of skeletal muscle differentiation in the 2D cell cultures and 3D organoids. Feature plots showing expression of muscle markers across across integrated scRNA-seq data from 'wild-type' 2D cultures (GSM4431314 in GSE147457; Chal et al. (2015) [15]) protocol in Xi et al. (2020) [50]) and 3D organoids (GSM4432416 in GSE147514, Mavrommatis et. al (2023) [18] protocol). The analysis of the datasets was performed as described in Kindler et al. (2025) [19] using seurat integrated analysis applying filter criteria as well as a regression step for cell cycle genes, total counts and stress genes according to [19]. Markers displayed in order: PAX7 (myogenic progenitor/ satellite cell identity), MYOD1 (myoblast determination), MYOG (terminal differentiation), MYH3 (embryonic myosin), MYH8 (neonatal myosin), DES (desmin, structural integrity).

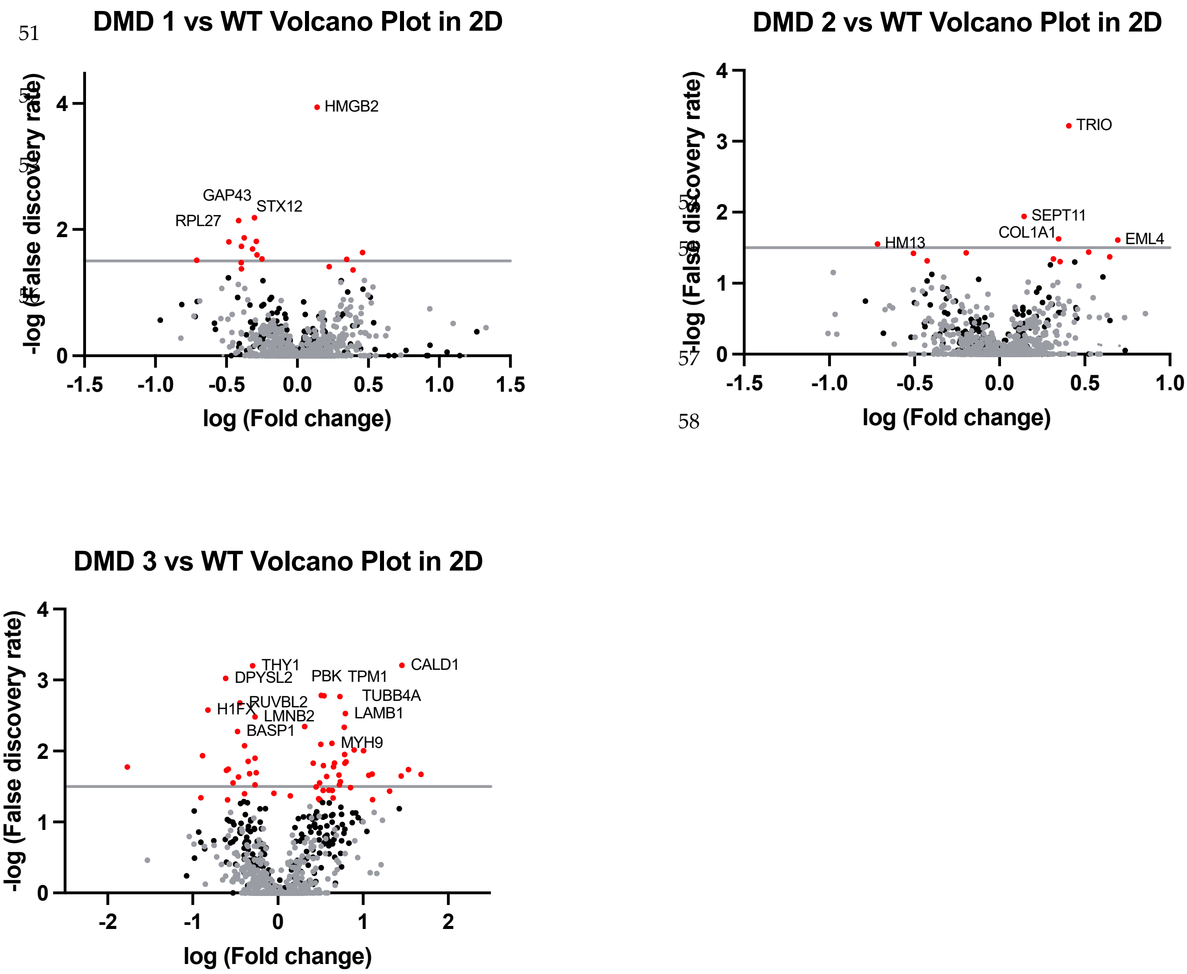

Figure S2 Results of the quantitative comparison of proteins after 2D myogenic differentiation of DMD1, DMD2, DMD3 patient and WT iPSC lines. Volcano plot displaying $-\log_{10}$-transformed *p*-values and $\log_2$-transformed fold changes (FC) of proteins matching the criteria for the quantitative comparison. In grey non-significant proteins, in black ANOVA significant and in red posthoc and ANOVA significant proteins. Proteins with highest Euclidean distances on both sides are indicated with their respective gene symbols.

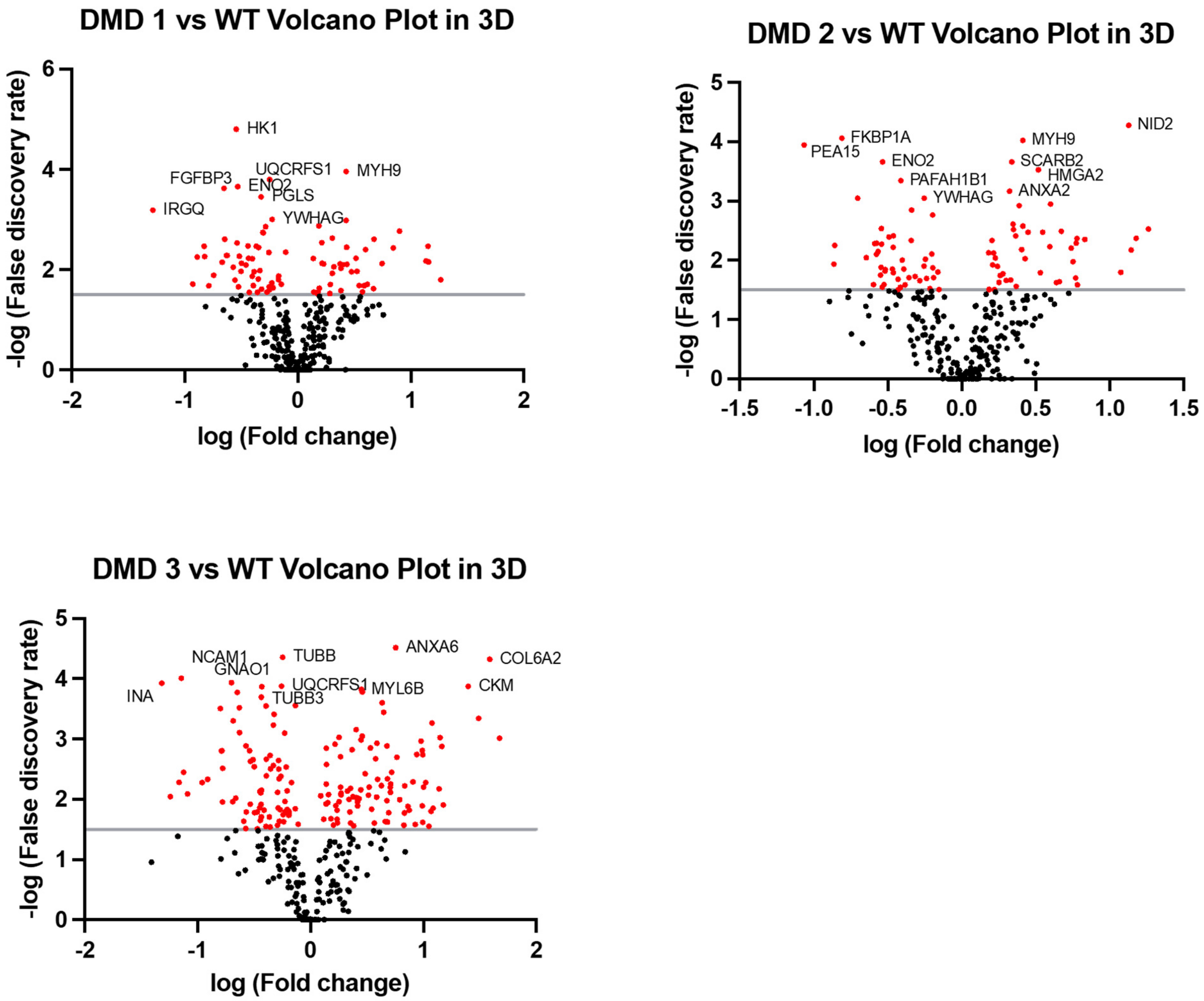

Figure S3 Results of the quantitative comparison of proteins after 3D skeletal muscle organoid differentiation of DMD1, DMD2, DMD3 patient and WT iPSC lines. Volcano plot displaying $-\log_{10}$-transformed *p*-values and $\log_2$-transformed fold changes (FC) of proteins matching the criteria for the quantitative comparison. In grey non-significant proteins, in black ANOVA significant and in red posthoc and ANOVA significant proteins. For a better overview in 3D non-significant proteins were excluded. Proteins with highest Euclidean distances on both sides are indicated with their respective gene symbols.
